# Optimized Liquid Medium Formulation for *Sanghuangporus vaninii* and Biological Activity of the Exopolysaccharides

**DOI:** 10.3390/foods13223574

**Published:** 2024-11-08

**Authors:** Haichen Huang, Xiaomin Li, Qi Lu, Hui Xu, Huijuan Sun, Junli Zhang, Xiaoping Wu, Junsheng Fu

**Affiliations:** 1 College of Life Sciences, Fujian Agriculture and Forestry University, Fuzhou 350002, China; haichenhuang312@gmail.com (H.H.); lxm2651277345@163.com (X.L.); xuhui189500@163.com (H.X.); fjwxp@126.com (X.W.); 2 Mycological Research Center, Fujian Agriculture and Forestry University, Fuzhou 350002, China; 3 College of Marine Sciences, Fujian Agriculture and Forestry University, Fuzhou 350002, China; 18950372995@163.com; 4 Tibet Academy of Agricultural and Animal Husbandry Sciences, Lhasa 850030, China; m18898002012@163.com (H.S.); xzyyxh888@163.com (J.Z.)

**Keywords:** *Sanghuangporus vaninii*, extracellular polysaccharide, fermentation medium, antioxidant, anticancer

## Abstract

Aims: *Sanghuangporus vaninii* (*S. vaninii*) is a rare medicinal mushroom that is rich in polysaccharides, triterpenes, flavonoids, and other bioactive compounds. It has good potential development value. Methods and Results: We performed single factor experiments and Box-Behnken response surface methodology to optimize the liquid fermentation medium formulation for *S. vaninii* with mycelial biomass as the indicator. The in vitro antioxidant and anti-cancer capacity of the exopolysaccharides of *S. vaninii* were estimated. The optimal liquid fermentation media composition for the MS-4, MS-6, and MS-8 strains of *Sanghuangporus vaninii* consisted of 25.86 ± 0.068 g/L maltose, 7.3 ± 0.043 g/L yeast extract, and 0.71 ± 0.005 g/L dandelion powder. The average mycelial biomass of *S. vaninii* under optimal conditions was 12.61 g/L. The mycelial biomass of the *Sanghuangporus vaninii* strains in the optimized formulation was 109–191% higher than that obtained with the basic potato dextrose broth (PDB). The exopolysaccharides of *Sanghuangporus vaninii* exhibited an ABTS radical scavenging activity with an EC_50_ of 0.021 ± 0.017 mg/mL and a DPPH radical scavenging activity with an EC_50_ of 0.076 ± 0.043 mg/mL. In anti-cancer assays, these exopolysaccharides demonstrated an IC_50_ value of 1.98 ± 0.36 mg/mL against PC-3 human prostate cancer cells, indicating significant bioactivity, highlighting their potential as functional food ingredients. Conclusions: In this study, the formula of liquid fermentation of *S. vaninii* strains was optimized, which lays a theoretical foundation for increasing the yield of *S. vaninii* and its application in industry. Moreover, our data showed the clinical potential of the *S. vaninii* exopolysaccharides as antioxidants and anti-cancer drugs.

## 1. Introduction

*Sanghuangporus vaninii* (*S. vaninii*) is a large, perennial, edible, and medicinal mushroom, known for its rich bioactive compounds, which grows on living or fallen poplar trees. It is not only a source of medicinal value but also holds promise as a functional ingredient in the development of health-promoting foods and nutraceuticals. It belongs to the phylum Basidiomycota, class Agaricomycetes, order Hymenochaetales, and family Hymenochaetaceae [1,2]. *S. vaninii* is distributed in Northeast China, South Korea, Eastern Russia, and Japan. *S. vaninii* consists of a reddish-brown or gray-black cap with a bright yellow edge, chestnut brown colored surface of the germ tube, and a yellow band on the back edge [3,4]. The current research regarding *S. vaninii* is mainly focused on areas such as liquid fermentation [5], extraction methods, determination of intrinsic components, and antioxidant, anti-tumor [6], anti-hyperlipidemia [7], anti-lung cancer [8], immunomodulatory [9], and other pharmacological activities. These effects make *Sanghuangporus vaninii* a food additive capable of increasing the nutritional value and functionality of food. *S. vaninii* can be used to make deep-processed products such as wine, cookies, tea, medicinal materials and noodles, and has great market potential. There is great demand for the *Sanghuangporus vaninii* mycelium and its bioactive components in countries such as South Korea.

Previous studies have shown that several bioactive compounds of *Sanghuangporus vaninii*, including polysaccharides, have been associated with significant antioxidant, antibacterial, tumor inhibition, and blood lipid-lowering activities [10,11]. He et al. identified two yellow exopolysaccharides of *Sanghuangporus vaninii* with high antioxidant activities [12]. They used gel filtration, infrared analysis, GC/MS, and SEC/MALLS combined viscosity methods to purify and characterize the properties of exopolysaccharides, including relative molecular weights, structures, and antioxidant activities. Zhang et al. extracted the polysaccharide from *S. vaninii* (PFSV) by destroying the cell wall and showed that PFSV was a promising drug candidate for lipid regulation [13].

*S. vaninii* has emerged as a research hotspot in the field of biological anti-cancer treatment because of significant antioxidant and anti-cancer effects. The yield of *S. vaninii* is limited for commercial use because of complex physiological and environmental requirements and a long growth cycle [14,15]. Currently, the submerged fermentation technique is the primary method for growing medicinal fungi because it is simple, controllable, and can be modified for large- or small-scale requirements [16]. Liquid fermentation is suitable for large-scale production of fungal mycelia that is required to extract sufficient quantities of high quality polysaccharides, polypeptides, polyphenols, alkaloids, and other bioactive metabolites in a relatively short period of time [17,18,19]. The chemical composition and physiological properties of the mycelia obtained by liquid fermentation are similar to those obtained from agricultural cultivation [20]. Therefore, liquid submerged fermentation is the main method for extracting and studying the bioactive compounds of *Sanghuangporus vaninii*. Teng et al. used mycelium biomass as the main indicator to screen the *Sanghuangporus vaninii* culture medium through response surface methodology and initially explored the best culture medium formula, achieving a highest biomass yield of 2.73 g [21]. Therefore, appropriate nutritional conditions such as carbon, nitrogen source, and inorganic salt dosage are key to the success of liquid fermentation. It is particularly important to select an excellent liquid culture medium formula.

Few studies have investigated the extracellular polysaccharides from *Sanghuangporus vaninii* grown in liquid fermentation media. The majority of previous studies have focused on the crude polysaccharides obtained from fruiting bodies. Furthermore, fixed composition of the liquid fermentation culture formulation is not currently available. Therefore, in this study, we performed single factor experiments to optimize the liquid fermentation medium with biomass as an indicator using response surface methodology. We also analyzed the antioxidant and anticancer activities of the extracellular polysaccharides in the fermentation medium. Our aim was to provide a reference for the extraction of bioactive polysaccharides from the fermentation broth.

## 2. Materials and Methods

### 2.1. Test Strains of Sanghuangporus vaninii

MS-4, MS-6 and MS-8 were obtained from the Mycological Research Center of Fujian Agriculture and Forestry University, Fuzhou, Fujian Province, China.

### 2.2. Preparation of Culture Medium

The solid potato dextrose agar (PDA) medium, a common component in food microbiology, was prepared with 200 g potatoes (peeled), 20 g glucose, 2% agar, 5 g peptone, 1.5 g MgSO_4_, 2 g KH_2_PO_4_, 0.01 g vitamin B_1_, and 1 L deionized water. The basic potato dextrose broth (PDB) was prepared with 200 g potatoes (peeled), 20 g glucose, 5 g peptone, 1.5 g MgSO_4_, 2 g KH_2_PO_4_, 0.01 g vitamin B_1_, and 1 L deionized water.

### 2.3. Strain Activation and Shake Flask Culture

Strain activation: The strains of *Sanghuangporus vaninii* were cultured on a plate with solid PDA at 25 °C. The mycelia, once sufficiently grown, were stored for later use.

Shake flask culture: The mycelium on the strain activation plate was punched with a 7 mm diameter hole puncher. Five mushroom cakes (7 mm diameter) were then inoculated into PDB medium. Growth conditions: shaking speed of 160 rpm, grown at 25 °C in the dark for 15 days.

### 2.4. Estimation of Mycelial Biomass

After growth in the liquid medium for 15 days, vacuum filtration was used to separate the mycelium from the fermentation broth. The mycelium was washed and placed on a dry clean petri dish, dried in an oven maintained at 60 °C, and weighed.

### 2.5. Single Factor Experiments

Single factor experiments were performed by varying the carbon and nitrogen sources to determine the optimal liquid fermentation medium. These experiments were designed to optimize the nutritional content for the production of bioactive compounds, which can be directly applied in the fortification of food products with enhanced health benefits.

### 2.6. Single Factor Experiment for Carbon Sources

The effects of different carbon sources on the mycelial mass of *Sanghuangporus vaninii* were analyzed by altering the carbon source in the basic enriched PDB formulation. PDB medium without glucose was used as the control group (CK group). The following carbon sources—maltose, sucrose, glucose, fructose, xylose, mannitol, mannose, lactose, and galactose—were utilized, each at a concentration of 20 g/L.

### 2.7. Single Factor Experiment for Nitrogen Sources

The effects of different nitrogen sources on the mycelial biomass of *Sanghuangporus vaninii* were analyzed by altering the nitrogen source in the basic enriched PDB formulation. PDB medium without peptone was used as the control group, and yeast extract, peptone, ammonium tartrate, ammonium sulfate, ammonium nitrate, urea, and beef extract were used as the nitrogen source in various experimental groups. Each nitrogen source was tested at a concentration of 5 g/L.

### 2.8. Screening the Optimal Dosage for the Carbon Source

Based on the carbon source screening results in the single-factor optimization experiment, different concentrations (10, 15, 20, 25, and 30 g/L) of the optimal carbon source were tested (in triplicate per group) to determine the concentration with the highest mycelial weight.

### 2.9. Screening the Optimal Dosage of the Nitrogen Source

Based on the nitrogen source screening results in the single-factor optimization experiment, different doses (2.5, 5, 7.5, 10, and 15 g/L) of the optimal nitrogen source were tested (in triplicate per group) to determine the concentration with the highest mycelial weight.

### 2.10. Screening the Optimal Dosage of the Exogenous Growth Factor

Preliminary experiments showed that addition of the dandelion powder significantly increased the mycelial weight of *Sanghuangporus vaninii*. Therefore, different concentrations of the dandelion powder (0, 0.5, 0.75, 1, 1.25, and 1.5 g/L) were added to the basic fermentation medium to determine the optimal concentration (3 replicates per group). At the end of culturing, the mycelial biomass was measured. The culture conditions and mycelial measurement methods were as described in Section 2.4.

### 2.11. Response Surface Optimization Experiment Using the Box-Behnken Design

Response surface analysis was performed using the Box-Behnken central combination design [22] with the optimized concentrations of carbon and nitrogen sources and the exogenous growth factor. The optimal carbon source, optimal nitrogen source, and dandelion powder were used as independent variables to perform a 3-factor and 3-level test with low, medium, and high concentrations for each independent variable. The mycelial biomass was the response value for optimal design and analysis.

### 2.12. Validation Test of the Response Surface Method

The media with the best predicted combination of multiple components was used to validate the results from the response surface method using mycelial biomass as the response variable. The best fermentation conditions were predicted by the response surface software.

### 2.13. Preparation and Purification of Extracellular Polysaccharides

After the completion of growth for 15 days, the fermentation broth was vacuum filtered and centrifuged at 10,000 rpm for 3 min. It was rotary evaporated and concentrated to 1/5th of the original volume. Then, the concentrated solution was mixed with four volumes of ethanol and allowed to precipitate at 4 °C overnight. The precipitate was harvested after centrifugation, re-dissolved with water, and deproteinized using the Sevag method [12]. Sevag reagent is a mixed solvent of chloroform and n-butanol in a ratio of 4:1. Subsequently, impurities were removed by extracting with ether and methanol. The purified extract was dialyzed against running water for 48 h. The polysaccharide samples were freeze-dried and stored at −20 °C for later use.

### 2.14. Determination of Antioxidant Capacity of the Extracellular Polysaccharides

Determination of the 2,2′-Azino-bis(3-ethylbenzothiazoline-6-sulfonic acid) or ABTS free radical scavenging ability: In the method referred to in the literature [23], extracellular polysaccharide solutions of different concentrations (0.025, 0.05, 0.25, 0.5, 1, 2 and 5 mg/mL) were used as the experimental group. Vitamin C is a widely recognized antioxidant, and a Vc solution of the same concentration gradient was used as the control group. ABTS solution was added to all the experimental and control wells at a ratio of 1:1. In the blank group, distilled water was used instead of the polysaccharide solution. The plate was incubated at 25 °C for 20 min. Then, the absorbance was measured at 734 nm in a microplate reader. ABTS free radical scavenging rate (%) = [1 − (A_x_ − A_x0_)/A_0_] × 100, where A_x_ is the absorbance value of the sample, A_x0_ is the absorbance value when distilled water is used instead of ABTS, and A_0_ is the absorbance value when distilled water is used instead of the sample.

Determination of the 2,2-diphenyl-1-picrylhydrazyl or DPPH free radical scavenging ability: In the method referred to in the literature [24], extracellular polysaccharide solutions of different concentrations (0.025, 0.05, 0.25, 0.5, 1, 2 and 5 mg/mL) were used as the experimental group. A Vc solution of the same concentration gradient was used as the control group. DPPH solution was added in a 1:1 ratio. In the blank group, an equal volume of absolute ethanol was added instead of the sample solution. The solutions were mixed well and incubated at 25 °C for 20 min. The absorbance value was then measured at 517 nm in a microplate reader. DPPH free radical scavenging rate (%) = [1 − (A_x_ − A_x0_)/A_0_] × 100, where A_x_ is the absorbance value of the sample, A_x0_ is the absorbance value of sample where 95% ethanol solution was used instead of DPPH, and A_0_ is the absorbance value of the sample when distilled water was used instead of the polysaccharide solution.

Determination of total antioxidant capacity: Total antioxidant capacity was estimated using the Ferric Reducing Antioxidant Power (FRAP) method as described by Li et al. [25]. We add 30 µL of the polysaccharide sample solutions with different concentrations and 180 µL of the FRAP working solution (0.3 mol/L pH3.6 sodium acetate buffer, 10 mmol/L TPTZ solution, and 20 mmol/L FeCl_3_ solution in a ratio of 10:1:1, prepared just before use) into the wells of a 96-well plate. Five replicates were used for each group. The plate was incubated at 25 °C for 20 min. The absorbance was measured at 593 nm in a microplate reader. As control, the polysaccharide sample was replaced with a standard solution of FeSO_4_ at a concentration range between 0.1~2.0 mmol/L. The standard curve was plotted with the concentration as the abscissa and absorbance as the ordinate. The regression equation was as follows: y = 0.4839 × −0.2152, correlation coefficient R² = 0.9991. The absorbance (A) value for each reaction was measured as A value = A_x_ − A_0_ − A_x0_, where A_x_ is the absorbance value of the sample, A_x0_ is the absorbance value of the sample with 95% ethanol solution instead of DPPH, and A_0_ is the absorbance value of the sample with distilled water instead of the sample. Then, the adjusted absorbance value (A) for each reaction was plotted on the standard curve and the corresponding FeSO_4_ concentration (mmol/L) was calculated to define the FRAP value. Larger FRAP value indicated stronger antioxidant activity.

### 2.15. Determination of Anti-Cancer Activity of the Extracellular Polysaccharides

Our method refers to and slightly modifies the method of Liu et al. [26]. The effect of exopolysaccharide on the viability of cancer cells was tested using well-growing cancer cell lines obtained from the American Type Culture Collection (ATCC), including HepG-2 (liver cancer), HCT-116 (colorectal cancer), MCF-7 (breast cancer), PC-3 (prostate cancer), U251 (glioblastoma), and T98G (glioblastoma multiforme). The cell suspension was diluted to a cell density of 1 × 10^4^ cells/mL, after which 100 μL was added into each cell of a 96-well plate. In the experimental group, cancer cells were incubated in medium containing different concentrations (0.025, 0.05, 0.25, 0.5, 1, 2 and 4 mg/mL) of the extracellular polysaccharides from three different strains of *Sanghuangporus vaninii*. In the control group, cancer cells were incubated with the complete culture medium without any extracellular polysaccharides. The blank group contained cell culture medium without cells. The plate was incubated for 24 h. Then, the culture medium was discarded and 180 μL complete culture medium containing 20 μL MTT solution (5 mg/mL) was added to each well. The plate was incubated for a further 4 h. Subsequently, the medium was discarded and 150 μL DMSO was added to each well and mixed thoroughly. The absorbance was analyzed at 490 nm in a microplate reader.

### 2.16. Statistical Analysis

SPSS software (ver. 26.0) and GraphPad Prism (ver. 5.0) software were used for the statistical analysis. The differences between groups were analyzed using one-way analysis of variance (ANOVA) and the LSD test. *p* < 0.05 indicated significant difference. *p* < 0.01 indicated highly significant difference.

## 3. Results

### 3.1. Effects of Different Carbon Sources on the Mycelial Biomass of Sanghuangporus vaninii

**Figure 1:**
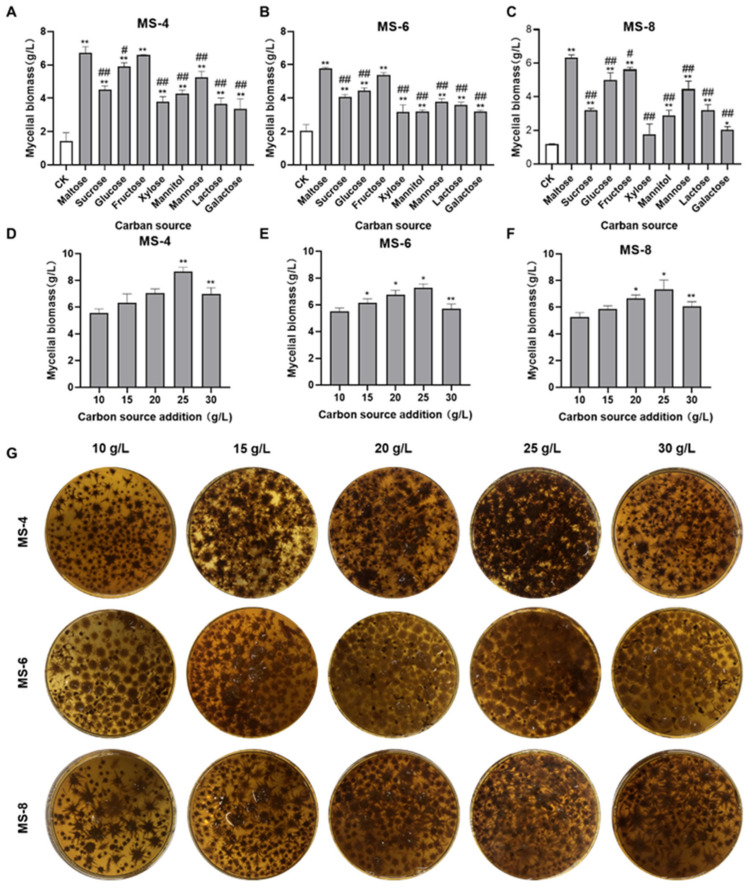
The effects of different carbon sources on the mycelial biomass of *Sanghuangporus vaninii*. (**A**–**C**) The effect of different carbon sources on the mycelial biomass of (**A**) MS-4, (**B**) MS-6, and (**C**) MS-8. (**D**–**F**) The effect of different concentrations of maltose on the mycelial biomass of (**D**) MS-4, (**E**) MS-6, and (**F**) MS-8. (**G**) The effect of different concentrations of maltose in the liquid fermentation broth on the spherical morphology and mycelial biomass of *Sanghuangporus vaninii*. Note: In the carbon source screening experiment, * p < 0.05 and ** p < 0.01 compared with the CK group; *# p* < 0.05 and *## p* < 0.01 compared with the optimal carbon source. In the optimal carbon source concentration screening experiment, * p < 0.05 and ** p < 0.01 compared with the previous group.

As shown in Figure 1A–C, maltose and fructose significantly increased the mycelial biomass of the three strains (MS-4, MS-6, and MS-8) of *Sanghuangporus vaninii*. Glucose and mannose can increase the mycelial biomass of the MS-4, MS-6, and MS-8 strains of *Sanghuangporus vaninii*. Xylose and galactose did not increase the mycelial biomass of MS-8. The overall results showed that maltose was the best carbon source for improving the mycelial biomass of *S. vaninii.* When grown in media containing maltose as the carbon source, the mycelial biomass was 6.74 g/L for MS-4, 6.74 g/L for MS-6, and 6.33 g/L for MS-8. Therefore, maltose was selected as the optimal carbon source for growing *Sanghuangporus vaninii*.

As shown in Figure 1D–G, we observed maltose concentration-dependent increase in the mycelial biomass of *Sanghuangporus vaninii.* The mycelial biomass was largest in liquid medium containing 25 g/L maltose. At this concentration, mycelial biomass was 8.67 g/L for MS-4, 7.26 g/L for MS-6, and 7.33 g/L for MS-8, which was 1.22-, 1.08-, and 1.1-fold higher than the mycelial biomass obtained with PDB medium containing 20 g/L maltose. The mycelial biomass decreased in liquid medium containing maltose concentrations > 25 g/L. Therefore, 25 g/L maltose was selected as the optimal concentration.

### 3.2. Effects of Different Nitrogen Sources on the Mycelial Biomass of Sanghuangporus vaninii

**Figure 2:**
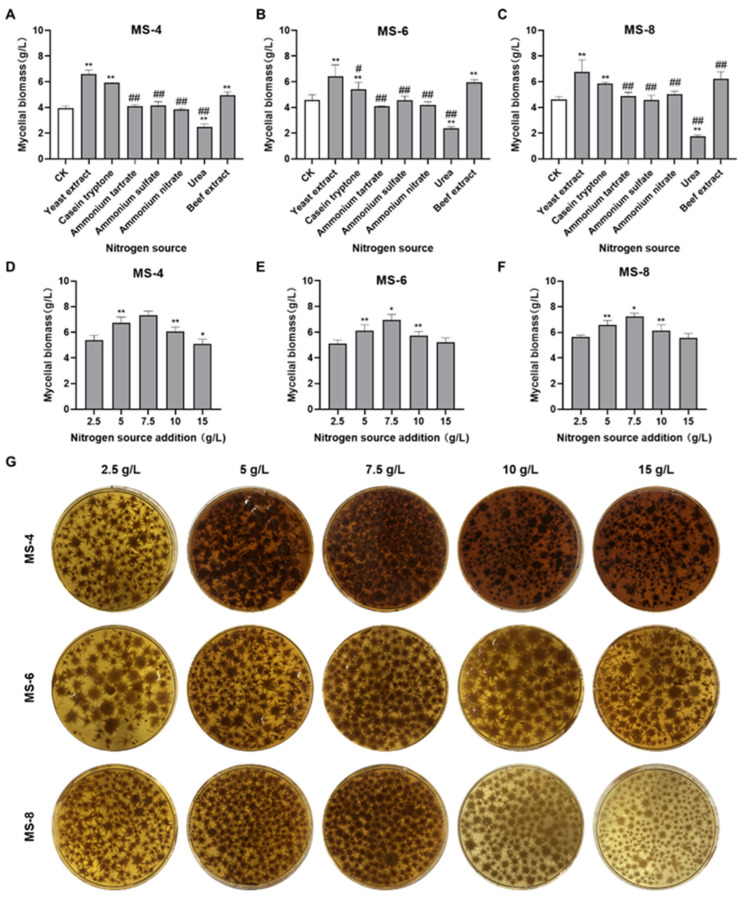
The effects of different nitrogen sources on the mycelial biomass of Sanghuangporus vaninii. (**A**–**C**) The effects of different nitrogen sources on the mycelial biomass of (**A**) MS-4, (**B**) MS-6, and (**C**) MS-8. (**D**–**F**) The effects of different concentrations of the yeast extract on the mycelial biomass of (**D**) MS-4, (**E**) MS-6, and (**F**) MS-8. (**G**) The effects of different concentrations of the yeast extract in the fermentation broth on the spherical morphology and mycelial biomass of *Sanghuangporus vaninii*. Note: In the nitrogen source screening experiment, * p < 0.05 and ** p < 0.01 compared with the CK group; *# p* < 0.05 and *## p* < 0.01 compared with the optimal nitrogen source. In the optimal nitrogen source dosage screening experiment, * p < 0.05 and ** p < 0.01 compared with the previous group.

As shown in Figure 2A–C, three nitrogen sources (yeast extract, peptone, and beef extract) significantly increased the mycelial biomass of *S. vaninii*. Among them, yeast extract was the best mycelial growth-promoting nitrogen source. Ammonium tartrate, ammonium sulfate, and ammonium nitrate increased the mycelial growth of MS-8 slightly but did not significantly increase the mycelial growth of MS-4 and MS-6 (*p* > 0.05). Urea did not increase the mycelial growth of *S. vaninii.* In liquid medium with yeast extract as the nitrogen source, the mycelial biomass was 6.59 g/L for MS-4, 6.43 g/L for MS-6, and 6.75 g/L for MS-8. Therefore, yeast extract was selected as the most suitable nitrogen source for the liquid fermentation medium to grow *S. vaninii*.

As shown in Figure 2D–G, we observed concentration-dependent increase in the mycelial biomass of *Sanghuangporus vaninii* with increasing concentrations of yeast extract. The mycelial biomass was largest in liquid medium containing 7.5 g/L yeast extract. At this concentration, the mycelial biomass was 7.33 g/L for MS-4, 6.97g/L for MS-6, and 7.23 g/L for MS-8. The mycelial growth declined in media containing > 7.5 g/L yeast extract. The mycelial biomass was 1.08 to 1.13-fold higher in liquid medium containing 7.5 g/L yeast extract compared with the liquid medium containing 5 g/L yeast extract. Therefore, the optimal concentration of yeast extract in the liquid fermentation medium for growing *Sanghuangporus vaninii* was 7.5 g/L.

### 3.3. Optimization of Exogenous Growth Factor Concentration in the Liquid Fermentation Medium

**Figure 3:**
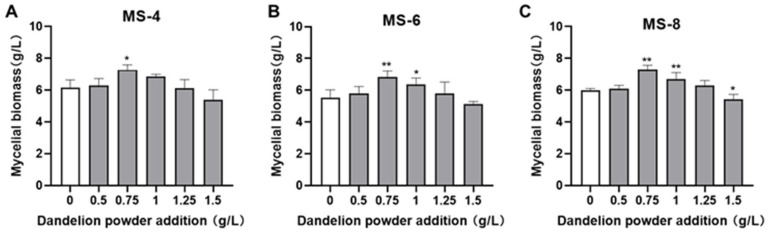
The effect of dandelion powder concentration on the mycelial biomass of *Sanghuangporus vaninii.* (**A**–**C**) The effect of different concentrations of the dandelion powder on the mycelial biomass of (**A**) MS-4, (**B**) MS-6, and (**C**) MS-8. Note: * *p* < 0.05 and ** *p* < 0.01 compared with the blank group.

As shown in Figure 3, addition of 0.75 g/L dandelion powder to the liquid fermentation broth significantly increased the mycelial growth of *S. vaninii*. The mycelial biomass in medium with 0.75 g/L dandelion powder was 1.18~1.23-fold greater than the blank group. However, higher concentrations of dandelion powder did not increase mycelial growth further. Therefore, 0.75 g/L of dandelion powder was selected as the optimal concentration for addition in the liquid fermentation medium to grow *S. vaninii*.

### 3.4. Response Surface Experimental Design and Results for S. vaninii Strains

#### 3.4.1. Response Surface Experimental Design and Results for the MS-4 Strain

The response surface experimental results for the MS-4 strain are shown in Table 1. The following fitted quadratic polynomial equation was generated for the MS-4 strain based on the mycelial biomass as the response value: Y = 13.512 + 0.6A − 0.24125B − 0.59125C − 0.335AB − 0.15AC + 0.0175BC − 1.79225A^2^ − 2.17475B^2^ − 2.00975C^2^. The variance and statistical significance of the quadratic polynomial equation in the response surface analysis are shown in Table 2. The model was statistically significant with a *p* value of <0.0001. The model terms (A, B, C, AB, A^2^, B^2^, and C^2^) were also significant with a *p* value of <0.05. The lack of fit (F) value was 1.34 and was not significantly different from pure error (*p* = 0.3789). Therefore, this model can be used to predict the optimal liquid fermentation media composition for increasing the mycelial biomass of MS-4.

**Figure 4:**
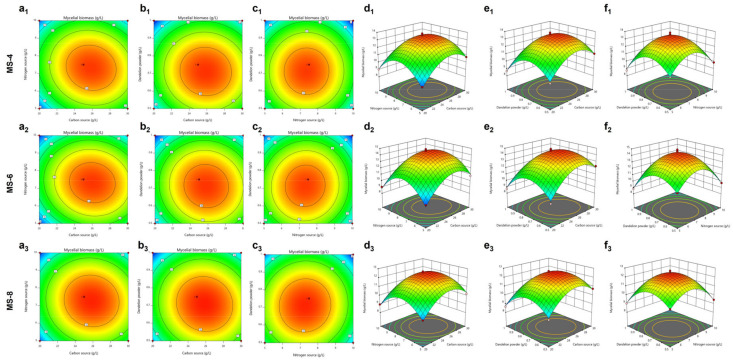
Response surface methodology using the Box-Behnken design to optimize the liquid fermentation medium for culturing *Sanghuangporus vaninii*. (**a**–**c**) Isoline values for various variables. (**d**–**f**) Interactions between different concentrations of (**d**) carbon sources and nitrogen sources, (**e**) carbon sources and dandelion powder, and (**f**) nitrogen sources and dandelion powder in the liquid fermentation medium.

The response surface based on the regression quadratic polynomial equation shows the interactions between the three variables (carbon source, nitrogen source, and dandelion powder) and their effect on the mycelial growth (Figure 4a_1_–f_1_). The interactions between the carbon and nitrogen sources significantly affected mycelial growth of *S. vaninii*. The predicted liquid fermentation medium composition for MS-4 contains 25.89 g/L of the carbon source, 7.32 g/L of the nitrogen source, and 0.71 g/L of dandelion powder. The predicted mycelial biomass of MS-4 under these conditions was 13.62g/L.

#### 3.4.2. Response Surface Experiment Design and Results for the MS-6 Strain

The response surface experimental results for the MS-6 strain are shown in Table 3. The following fitted quadratic polynomial equation was generated based on the MS-6 mycelial biomass as the response value: Y = 14.474 + 0.81875A − 0.19875B − 0.67C − 0.5575AB − 0.245AC + 0.185BC − 2.383A^2^ − 2.723B^2^ − 2.285C^2^. The variance and statistical significance of the quadratic polynomial in the response surface analysis are shown in Table 4. The model was statistically significant with a *p* value of <0.0001. The important model terms were A, C, AB, A^2^, B^2^, and C^2^. The lack of fit (F) value was 0.7893 and was not significantly different from the pure error (*p* = 0.5596). Therefore, this model can be used to predict the optimal liquid fermentation composition to increase the mycelial biomass of MS-6.

The response surface generated based on the regression quadratic polynomial showed the interactions between the three variables (carbon source, nitrogen source, and dandelion powder) and their effect on the mycelial biomass of MS-6 (Figure 4a_2_–f_2_). The interaction between the carbon and nitrogen sources was important for mycelial growth. The predicted optimal liquid fermentation composition for growing *S. vaninii* based on the response surface model included 25.93 g/L of the carbon source, 7.34 g/L of the nitrogen source, and 0.71 g/L of the dandelion powder. The mycelial biomass of MS-6 was expected to be 14.6 g/L under these predicted growth conditions.

#### 3.4.3. Response Surface Experiment Design and Results for the MS-8 Strain

The response surface experimental results for the MS-8 strain are shown in Table 5. The following fitted quadratic polynomial equation was generated for the MS-8 strain with mycelial biomass as the response value: Y = 12.512 + 0.39625A − 0.28B − 0.55625C − 0.175AB − 0.3325AC + 0.1BC-1.552A^2^ − 1.5547B^2^ − 1.6125C^2^. The quadratic polynomial variance and statistical significance are shown in Table 6. The model was statistically significant (*p* < 0.0001). In this model, A, B, C, AB, AC, A^2^, B^2^, C^2^ were important model terms. The lack of fit (F) value was 0.34 and was not significantly different from the pure error (*p* = 0.8020). Therefore, this model can be used to predict the optimal liquid fermentation medium composition to increase the mycelial biomass of the MS-8 strain.

The response surface model generated for the MS-8 strain based on the regression quadratic polynomial equation (Figure 4a_3_–f_3_) showed interactions between the three variables (carbon source, nitrogen source, and dandelion powder) and their effects on the MS-8 mycelial biomass. The interactions between carbon and nitrogen sources as well as carbon sources and dandelion powder were important. The predicted optimal liquid fermentation composition based on the response surface model for the MS-8 strain included 25.77 g/L of the carbon source, 7.24 g/L of the nitrogen source, and 0.70 g/L of the dandelion powder. The mycelial biomass of MS-8 was predicted to reach 12.61 g/L under these optimal conditions.

### 3.5. Verification Results of the Optimal Fermentation Medium for Sanghuangporus vaninii

**Figure 5:**
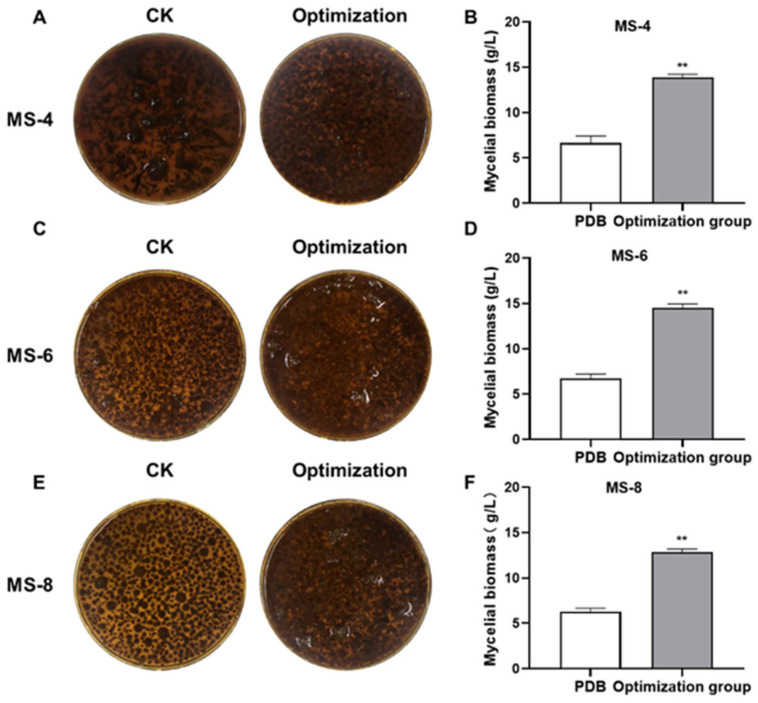
Validation experiment results for the liquid fermentation optimization formula. (**A**,**C**,**E**) Spherical morphology of *Sanghuangporus vaninii* in PDB medium and optimized fermentation medium formula. (**B**,**D**,**F**) Mycelial biomass of *Sanghuangporus vaninii* in PDB medium and optimized fermentation medium formula. Note: ** *p* < 0.01 compared with the PDB group.

The predicted liquid fermentation culture conditions were then verified by growing the three different strains of *S. vaninii* with the optimized media. Subsequently, the mycelium was collected, dried, and weighed. The experimental results are shown in Figure 5. The MS-4 mycelial weight in the control PDB group was 6.63 ± 0.77 g/L compared to 13.88 ± 0.34 g/L (2.09-fold increase) in the optimized PDB group. The predicted weight of the MS-4 mycelial biomass was 13.62 g/L (Figure 5A,B). The MS-6 mycelial weight in the control PDB group was 6.73 ± 0.47 g/L compared with 14.48 ± 0.46 g/L (2.15-fold increase) in the optimized fermentation medium. The predicted weight of the MS-6 strain was 14.6 g/L (Figure 5C,D). The MS-8 mycelial biomass in the control PDB medium was 6.7 ± 0.36 g/L compared with 12.82 ± 0.37 g/L (2.91-fold increase) in the optimized fermentation medium. The predicted MS-8 mycelial biomass was 12.61 g/L (Figure 5E,F). These data validated the prediction model.

### 3.6. Estimation of the Antioxidant Activities of Extracellular Polysaccharides from the S. vaninii Strains

**Figure 6:**
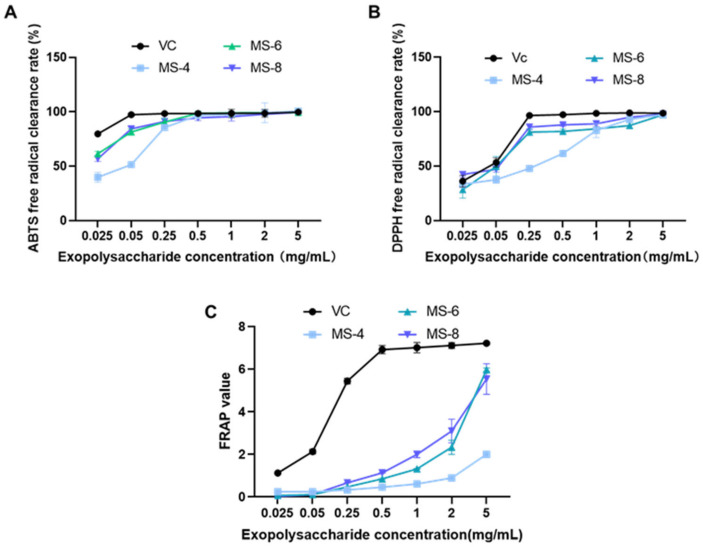
Antioxidative activity of the exopolysaccharides isolated from the culture media of *Sanghuangporus vaninii*. (**A**) ABTS radical scavenging ability; (**B**) DPPH radical scavenging ability; (**C**) total antioxidant capacity.

The chemical antioxidant activities of the extracellular polysaccharides from the MS-4, MS-6, and MS-8 strains of *S. vaninii*, which could be instrumental in developing food products with improved nutritional profiles, are shown in Figure 6. The EC_50_ values represent the concentration of exopolysaccharides required to scavenge 50% of free radicals. The free radical scavenging activities of the extracellular polysaccharides from the three strains of *S. vaninii* were highly significant compared with the control. Furthermore, the scavenging effect on the ABTS free radicals (EC_50_ = 0.021 ± 0.017 mg/mL) was higher than the scavenging effect on the DPPH free radicals (EC_50_ = 0.076 ± 0.043 mg/mL). Subsequently, we analyzed the total antioxidant capacity of the extracellular polysaccharides isolated from the three strains. Extracellular polysaccharides from the MS-6 (EC_50_ = 0.012 mg/mL) and the MS-8 (EC_50_ = 0.011 mg/mL) strains showed higher antioxidant capacity compared with that from the MS-4 (EC_50_ = 0.041 mg/mL) strain in the ABTS free radical scavenging experiment. In the DPPH free radical scavenging experiment, the scavenging activities of the extracellular polysaccharides from the MS-6 (EC_50_ = 0.062 mg/mL) and the MS-8 (EC_50_ = 0.043 mg/mL) strains were comparable and stronger than those from the MS-4 strain (EC_50_ = 0.125 mg/mL). FRAP assay results showed that the total antioxidant capacity of the extracellular polysaccharides of the MS-6 (5.97 ± 0.08) and MS-8 (5.54 ± 0.72) strains were higher than those from the MS-4 strain (2.006 ± 0.08). The total antioxidant activity of the extracellular polysaccharides from the MS-4 strain was lower compared with the extracellular polysaccharides from the MS-6 and MS-8 strains.

### 3.7. Estimation of Anti-Cancer Activities of Extracellular Polysaccharides from the S. vaninii Strains

**Figure 7:**
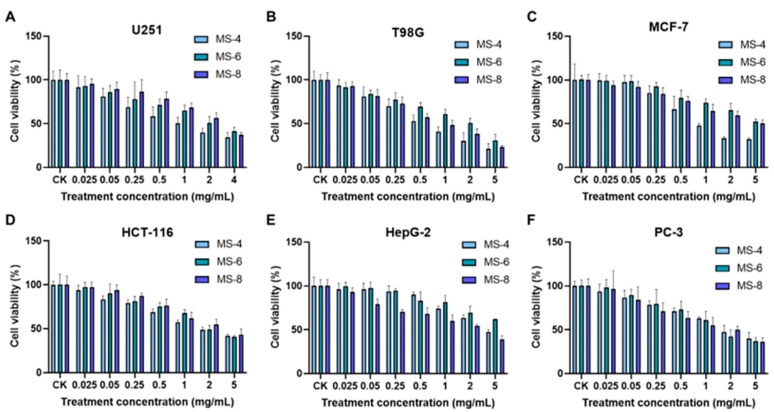
The effects of extracellular polysaccharides of *Sanghuangporus vaninii* on the in vitro growth of (**A**) U251 human glioma cells; (**B**) T98G human glioblastoma cells; (**C**) MCF-7 human breast cancer cells; (**D**) HCT-116 human colon cancer cells; (**E**) HepG2 human hepatoma cells; (**F**) PC-3 human prostate cancer cells.

The in vitro anti-cancer activities of the exopolysaccharides from the three strains of *S. vaninii* (MS-4, MS-6, and MS-8) were analyzed against six different types of cancer cells (Figure 7). The IC_50_ value refers to the concentration of exopolysaccharides required to reduce cell viability by 50%. The fungal exopolysaccharides significantly affected the viability of the six types of cancer cells, especially T98G human glioblastoma cells (IC_50_ = 1.104 ± 0.64 mg/mL) and PC3 human prostate cancer cells (IC_50_ = 1.98 ± 0.36 mg/mL). The exopolysaccharides from the MS-4 strain showed the highest cell death-inducing effects on the T98G cells. The survival rate of T98G cells was 21.06% when treated with the highest concentration (5 mg/mL) of MS-4 exopolysaccharides. The half effective concentration (IC_50_ value) of the MS-4 exopolysaccharides was 0.634 mg/mL. The highest dose of the MS-6 and MS-8 exopolysaccharides reduced the survival rate of the T98G cells to 30.95% and 23.02%, respectively. The corresponding IC_50_ values were 1.829 mg/mL and 0.849 mg/mL, respectively. These data demonstrated that the exopolysaccharides of *Sanghuangporus vaninii* were associated with significant cell death-inducing effects on the brain glioma cells (T98G). The survival rate of the PC-3 cells was 36.54% to 40.03% at the highest concentration of the exopolysaccharides from the MS-4, MS-6, and MS-8 strains. The IC_50_ value for the MS-8 exopolysaccharides was 1.684 mg/mL. This was higher compared with the MS-4 exopolysaccharides (IC_50_ = 2.384 mg/mL) and the MS-6 exopolysaccharides (IC_50_ = 1.895 mg/mL). The cell death-inducing effect of the exopolysaccharides was weaker in the HepG2 liver cancer cells. MS-8 exopolysaccharides (IC_50_ = 2.225 mg/mL) showed stronger cell death-inducing effects on the liver cancer cells than the MS-4 (IC_50_ = 5.14 mg/mL) and MS-6 (IC_50_ = 5 mg/mL) exopolysaccharides. Furthermore, MS-4 exopolysaccharides showed the strongest cell death-inducing effects on the MCF-7 human breast cancer cells compared with the MS-6 and MS-8 exopolysaccharides. The survival rate of MCF-7 cells was 32.35% when treated with the highest concentration (5 mg/mL) of the MS-4 exopolysaccharides. The half inhibitory concentration (IC_50_) of the MS-4 exopolysaccharides was 1.131 mg/mL. The cell death-inducing effect of the MS-6 and MS-8 exopolysaccharides was lower than that of the MS-4 exopolysaccharides. The survival rate of the MCF-7 cells was 52.46% and 50.46% when treated with 5 mg/mL of MS-6 and MS-8 exopolysaccharides, respectively. Exopolysaccharides from the three strains showed similar cell death-inducing effects on the HCT-116 colon cancer cells. When treated with 5 mg/mL exopolysaccharides, the survival rate of the HCT-116 cells ranged from 41.71% to 43.13%. The IC_50_ values were 2.247 mg/mL for the MS-4 exopolysaccharides, 2.767 mg/mL for the MS-6 exopolysaccharides, and 2.935 mg/mL for the MS-8 exopolysaccharides. In the U251 human glioma cells, MS-4 exopolysaccharides showed higher efficacy than the MS-6 and MS-8 exopolysaccharides. The survival rate of the U251 cells ranged between 34.56~41.26% with the highest dose. The IC_50_ values for the MS-4, MS-6, and MS-8 exopolysaccharides were 1.147 mg/mL, 3.016 mg/mL, and 2.785 mg/mL, respectively.

## 4. Discussion

Traditional solid-state fermentation technology cannot meet the growing industrial demands despite significant advances in the cultivation and downstream processing technologies of edible and medicinal mushrooms. Therefore, liquid fermentation technology has gradually become the mainstream technology for the industrial production of the edible and medicinal mushroom [27,28]. The liquid fermentation medium requires basic components such as carbon source, nitrogen source, inorganic salts, and vitamins. Some fungal strains require addition of some exogenous compounds or growth factors to increase the mycelial biomass [29]. Previous study of the nutritional characteristics of *S. vaninii* showed that the carbon source, nitrogen source, and dandelion powder were essential components of the growth medium and influenced the mycelial weight. This study used response surface analysis to optimize the liquid fermentation culture medium for three strains of *S. vaninii*. The optimized fermentation medium formulation for the MS-4 strain included 25.89 g/L of maltose, 7.32 g/L of yeast extract, 0.71 g/L of dandelion powder, 1.5 g/L of MgSO_4_, 2 g/L of KH_2_PO_4_, 0.01 g/L of vitamin B_1_, and 1L of deionized water. The optimal fermentation medium formulation for the MS-6 strain included 25.93 g/L of maltose, 7.34 g/L of yeast extract, 0.71 g/L of dandelion powder, 1.5 g/L of MgSO_4_, 2 g/L of KH_2_PO_4_, 0.01 g/L of vitamin B_1_, and 1L of deionized water. The optimal fermentation medium formulation for the MS-8 strain included 25.77 g/L of maltose, 7.24 g/L of yeast extract, 0.7 g/L of dandelion powder, 1.5 g/L of MgSO_4_, 2 g/L of KH_2_PO_4_, 0.01 g/L of vitamin B_1_, and 1 L of deionized water. The predicted mycelial biomass with the optimized liquid fermentation medium was 13.62 g/L for MS-4, 14.6 g/L for MS-6, and 12.61 g/L for MS-8. The verification tests with the optimized liquid fermentation medium showed that the mycelial biomass for the MS-4, MS-6, and MS-8 strains increased by 109%, 115%, and 191%, respectively, compared with the control PDB medium. Therefore, the optimized effect was significant with high industrial application value. The nitrogen source experiments showed that organic nitrogen sources such as yeast extract, peptone, and beef extract significantly increased the mycelial growth of *S*. *vaninii*, whereas inorganic nitrogen sources such as urea were not effective in increasing mycelial growth. These data were consistent with the results previously reported by Zhong et al. [30].

Based on He’s research, we used the alcohol precipitation method to extract polysaccharides, followed by the Sevag method for protein removal to obtain crude polysaccharides, which were then subjected to further separation and purification. The study showed that the exopolysaccharides from *Sanghuangporus vaninii* includes ribose, xylose, glucuronic acid, galactose, glucose, mannose, and galacturonic acid [23]. We also measured the antioxidant activities of the exopolysaccharides from the culture medium of *S. vaninii.* The in vitro free radical scavenging ability of the exopolysaccharides increased proportionately with higher concentrations. These data were comparable with the findings reported by Wang et al. [31]. Exopolysaccharides from the three strains of *S. vaninii* showed significant free radical scavenging abilities. The free radical scavenging effect was higher for the ABTS free radicals (EC_50_ = 0.021 ± 0.017 mg/mL) compared with the DPPH free radicals (EC_50_ = 0.076 ± 0.043 mg/mL). Compared with the MS-4 and MS-6 exopolysaccharides, the MS-8 polysaccharides showed stronger ABTS free radical scavenging ability, with an EC_50_ value of 0.011 mg/mL. Moreover, MS-6 and MS-8 exopolysaccharides showed stronger DPPH free radical scavenging abilities than the MS-4 exopolysaccharides. Total antioxidant activities of the MS-6 and MS-8 exopolysaccharides were higher than those of the MS-4 exopolysaccharides.

The in vitro experiments also demonstrated that the exopolysaccharides significantly affected the viability of T98G human glioblastoma cells (IC_50_ = 1.104 ± 0.64 mg/mL) and PC-3 human prostate cancer cells (IC_50_ = 1.98 ± 0.36 mg/mL). The cell death-inducing effects of the MS-4 exopolysaccharides on the T98G cells were higher than those of the MS-6 and MS-8 exopolysaccharides. The survival rates of the T98G cells were 21.06%, 30.95%, and 23.02% when treated with the highest concentrations (5mg/mL) of the MS-4, MS-6, and MS-8 exopolysaccharides, respectively. The survival rate was 36.54~40.03% for the PC-3 cells, 32.35~52.46% for the MCF-7 cells, 41.71~43.13% for the HCT-116 cells, and 34.56~41.26% for the U251 cells. The cell death-inducing effects of the *S. vaninii* exopolysaccharides were weaker on the HepG2 liver cancer cells. Overall, *S. vaninii* exopolysaccharides showed significant anti-cancer effects on the six cancer cell lines tested, thereby providing evidence for the subsequent development of anti-cancer drugs using the *S. vaninii* exopolysaccharides.

The optimized production of exopolysaccharides rich in bioactive compounds positions *Sanghuangporus vaninii* as a candidate for the development of functional foods with targeted health benefits, such as enhanced immunity and cancer prevention.

## 5. Conclusions

In summary, this study used single factor experiments and response surface methodology to optimize the liquid fermentation media composition for three strains of *S. vaninii*. The optimal fermentation medium formulation included 25.86 ± 0.068 g/L of maltose, 7.3 ± 0.043 g/L of yeast extract, 0.71 ± 0.005 g/L of dandelion powder, 1.5 g/L of MgSO_4_, 2 g/L of KH_2_PO_4_, 0.01 g/L of vitamin B_1_, and 1L of deionized water. The mycelial biomass of the *S. vaninii* strains increased by 109–191% using the optimized media formulation compared with the basic PDB formulation. The exopolysaccharides from the three strains of *Sanghuangporus vaninii* showed significant in vitro antioxidant and anti-cancer activities. This demonstrated significant evidence for using *S. vaninii* to develop natural antioxidant products and anti-cancer drugs in the future. This study not only optimized the fermentation process for enhanced production of bioactive compounds from *S. vaninii* but also established a foundation for their application in the food industry, particularly in the development of functional foods with natural antioxidant properties and potential anti-cancer benefits.

## Figures and Tables

**Table 1:** Response surface design and test results of MS-4.

No.	A: Carbon Source (g/L)	B: Nitrogen Source (g/L)	C: Dandelion Powder (g/L)	Mycelial Biomass (g/L)
1	30	7.5	1	9.76
2	30	5	0.75	10.73
3	25	10	1	8.53
4	20	5	0.75	9.06
5	25	7.5	0.75	13.33
6	20	7.5	1	8.66
7	25	5	1	8.76
8	25	7.5	0.75	13.46
9	25	10	0.5	9.86
10	20	10	0.75	9.03
11	25	5	0.5	10.16
12	30	7.5	0.5	11.06
13	25	7.5	0.75	13.65
14	30	10	0.75	9.36
15	25	7.5	0.75	13.86
16	25	7.5	0.75	13.26
17	20	7.5	0.5	9.36

**Table 2:** Variance analysis and significance tests of MS-4.

Source	Sum of Squares	Df	Mean Squares	F-Value	*p*-Value
Model	63.01	9	7.00	101.95	<0.0001
A	2.88	1	2.88	41.94	0.0003
B	0.4656	1	0.4656	6.78	0.0352
C	2.80	1	2.80	40.72	0.0004
AB	0.4489	1	0.4489	6.54	0.0377
AC	0.0900	1	0.0900	1.31	0.2899
BC	0.0012	1	0.0012	0.0178	0.8975
A^2^	13.52	1	13.52	196.95	<0.0001
B^2^	19.91	1	19.91	289.98	<0.0001
C^2^	17.01	1	17.01	247.65	<0.0001
Residual	0.4807	7	0.0687		
Lack of fit	0.2412	3	0.0804	1.34	0.3789
Pure error	0.2395	4	0.0599		
Cor Total	63.49	16			

R^2^ = 0.9924; adjusted R^2^ = 0.9827; predicted R^2^ = 0.9333.

**Table 3:** Response surface design and test results of MS-6.

No.	A: Carbon Source (g/L)	B: Nitrogen Source (g/L)	C: Dandelion Powder (g/L)	Mycelial Biomass (g/L)
1	25	5	0.5	10.8
2	20	7.5	1	8.7
3	30	10	0.75	9.56
4	30	5	0.75	10.8
5	20	10	0.75	9.05
6	30	7.5	1	9.86
7	25	7.5	0.75	14.2
8	25	10	0.5	9.76
9	25	7.5	0.75	14.9
10	25	7.5	0.75	14
11	20	5	0.75	8.03
12	30	7.5	0.5	11.4
13	25	5	1	8.8
14	25	10	1	8.5
15	20	7.5	0.5	9.26
16	25	7.5	0.75	14.6
17	25	7.5	0.75	14.67

**Table 4:** Variance analysis and significance tests of MS-6.

Source	Sum of Squares	Df	Mean Squares	F-Value	*p*-Value
Model	97.03	9	10.78	88.52	<0.0001
A	5.36	1	5.36	44.03	0.0003
B	0.3160	1	0.3160	2.59	0.1513
C	3.59	1	3.59	29.49	0.0010
AB	1.24	1	1.24	10.21	0.0152
AC	0.2401	1	0.2401	1.97	0.2031
BC	0.1369	1	0.1369	1.12	0.3243
A^2^	23.92	1	23.92	196.36	<0.0001
B^2^	31.23	1	31.23	256.38	<0.0001
C^2^	22.00	1	22.00	180.62	<0.0001
Residual	0.8525	7	0.1218		
Lack of fit	0.3170	3	0.1057	0.7893	0.5596
Pure error	0.5355	4	0.1339		
Cor Total	97.88	16			

R^2^ = 0.9913; adjusted R^2^ = 0.9801; predicted R^2^ = 0.9396.

**Table 5:** Response surface design and test results of MS-8.

No.	A: Carbon Source (g/L)	B: Nitrogen Source (g/L)	C: Dandelion Powder (g/L)	Mycelial Biomass (g/L)
1	25	10	1	8.63
2	25	10	0.5	9.46
3	30	5	0.75	10.2
4	30	7.5	1	8.83
5	30	7.5	0.5	10.73
6	20	5	0.75	9.13
7	25	7.5	0.75	12.67
8	20	10	0.75	8.96
9	20	7.5	0.5	9.16
10	25	5	0.5	10.26
11	30	10	0.75	9.33
12	25	7.5	0.75	12.33
13	20	7.5	1	8.67
14	25	7.5	0.75	12.7
15	25	7.5	0.75	12.46
16	25	7.5	0.75	12.4
17	25	5	1	9.03

**Table 6:** Variance analysis and significance tests of MS-8.

Source	Sum of Squares	Df	Mean Squares	F-Value	*p*-Value
Model	39.96	9	4.44	228.54	<0.0001
A	1.26	1	1.26	64.65	<0.0001
B	0.6272	1	0.6272	32.28	0.0007
C	2.48	1	2.48	127.40	<0.0001
AB	0.1225	1	0.1225	6.30	0.0403
AC	0.4970	1	0.4970	25.58	0.0015
BC	0.0400	1	0.0400	2.06	0.1945
A^2^	10.15	1	10.15	522.16	<0.0001
B^2^	10.18	1	10.18	523.84	<0.0001
C^2^	10.94	1	10.94	563.31	<0.0001
Residual	0.1360	7	0.0194		
Lack of fit	0.0273	3	0.0091	0.3352	0.8020
Pure error	0.1087	4	0.0272		
Cor Total	40.10	16			

R^2^ = 0.9966; adjusted R^2^ = 0.9922; predicted R^2^ = 0.9849.

## Data Availability

The original contributions presented in the study are included in the article, further inquiries can be directed to the corresponding author.
